# The Relationship Between Healthy Lifestyle Behaviours and Levels of Health Literacy of University Students in Mogadishu, Somalia

**DOI:** 10.3390/ijerph22081259

**Published:** 2025-08-11

**Authors:** Simay Akyuz, Elif Donmez, Betülay Kılıç, Nasra Alı Jama, Hasan Hüseyin Eker

**Affiliations:** 1 Department of Oncology Nursing, Gulhane Faculty of Nursing, University of Health Sciences, Ankara 06010, Turkey; 2 Department of Oncology Nursing, Hamıdıye Faculty of Nursing, University of Health Sciences, Istanbul 34668, Turkey; 3 Department of Internal Medıcıne Nursing, Gulhane Faculty of Nursing, University of Health Sciences, Ankara 06010, Turkey; 4 Somalia Mogadishu Recep Tayyip Erdogan Faculty of Health Sciences, University of Health Sciences, Mogadishu 2526, Somalia

**Keywords:** healthy lifestyle behaviours, health literacy, students

## Abstract

According to the WHO, health problems in Somalia are reported to be at an alarming level today and in the future. Objective: The aim of this study was to determine the relationship between healthy lifestyle behaviours and the levels of health literacy of university students receiving health sciences education in Mogadishu, Somalia. Methods: This descriptive, cross-sectional study was conducted in April 2024 in a university delivering education in Turkish in Mogadishu. The study sample comprised 219 health sciences students. The data collection tools used were a demographic data form, the Healthy Lifestyle Behaviours Scale II (HLBS-II) and the Turkish version of the European Health Literacy Scale (HLS-EU-TR). Results: A total of 219 students participated, with 86.3% identifying as female and 13.7% as male, and the average age was 20.91 ± 2.2 years. The mean of the total scores obtained for the HLBS II was found to be 127.54 ± 23.46 and the mean of the total scores obtained for HLS-EU-TR was 30.33 ± 8.17, while scores across all dimensions of the HLS-EU-TR indicated a problematic−borderline level. Analysis showed that with each advancing year of study, there was a statistically significant increase in health responsibility, physical activity, nutrition and total HLBS scores (*p* < 0.05). A positive correlation was observed between healthy lifestyle behaviours and HLS-EU-TR total scores, with correlation coefficients as follows: r = 0.230, *p* = 0.001; r = 0.215, *p* = 0.001; r = 0.193, *p* = 0.004; r = 0.308, *p* < 0.001; r = 0.247, *p* < 0.001; r = 0.284, *p* < 0.001; r = 0.313, *p* < 0.001. Furthermore, 13.1% of the change in healthy lifestyle behaviours was explained by the HLS-EU-TR Treatment and Services subdimension and grade level (R^2^ = 0.131). The HLS-EU-TR Treatment and Services subdimension and grade level positively contributed to the level of healthy lifestyle behaviours (ß = 0.373; ß = 0.164). Conclusion: It can be concluded that the identification of a positive correlation between health literacy and healthy lifestyle behaviours indicates that health literacy significantly influences healthy lifestyle choices. This correlation suggests that as students progress through their educational journey, their knowledge and behaviours toward health improve, highlighting that increased educational attainment equips individuals with the skills necessary to engage more effectively in the healthcare system and to translate acquired knowledge into behaviour. These findings underscore the critical role of ongoing health education initiated from an early age.

## 1. Introduction

The term “healthy lifestyle behaviours” encompasses a series of behaviours that directly affect the health of an individual. These behaviours include personal habits such as physical activity, a balanced diet, sufficient sleep and stress management and are the basic components of a lifestyle shaped by the individual’s cultural background, religious beliefs, socioeconomic status and personal perceptions [1,2].

According to the World Health Organization (WHO) data, 60% of an individual’s quality of life and health status is directly associated with their lifestyle and behaviours. Although it is traditionally accepted that the foundations for healthy lifestyle behaviours are laid in childhood and adolescence, the age range of 18–30 years and university life have emerged as a critical period for reinforcing these behaviours [3].

Studies reveal that a significant proportion of college students lead risky lifestyles and tend to engage in behaviours that negatively impact their health, rather than adopting health-improving behaviours [4]. A study conducted on 800 university students in Iran supports this. In the study, it was found that approximately 13.3% of the students smoked regularly, 14.3% consumed alcohol at least once, and excessive alcohol use occurred every day. Although 95% of the students reported engaging in regular physical activity, it was determined that their eating habits primarily consisted of unhealthy options, such as fast food and high-sodium content, and only 23.9% of them had a normal body weight [5]. Similarly, a study conducted among Saudi university students revealed that overweight and obesity are alarmingly common. Unhealthy lifestyle habits, dietary patterns and psychological factors were found to contribute to this condition [6]. Another study on first-year college students in China found that unhealthy lifestyle behaviours, particularly caffeinated beverage consumption and prolonged screen time, were negatively associated with sleep quality [7].

A study examining the impact of a healthy lifestyle on mental health yielded remarkable findings. The study found that a healthy lifestyle is strongly associated with a reduced risk of mental disorders, depression, anxiety, bipolar disorder, lower mortality rates and longer life expectancy [8]. Unhealthy lifestyle behaviours observed in university students may increase risk factors for future diseases and indirectly affect mortality rates [9]. Therefore, promoting healthy lifestyle behaviours during the university period is of great importance.

Studies examining the relationship between health behaviours and weight status in the literature show that at least 60 min of physical activity per day reduces the likelihood of being overweight or obese [10]. In addition, various measures to promote healthy lifestyle behaviours, such as health responsibility, physical activity, healthy eating and stress management, were presented to university students through a peer-supported e-health module aimed at protecting health and improving public health [11].

Individuals’ adoption of healthy lifestyle behaviours, effective utilization of health services and protection of their health are directly related to the level of health literacy [12]. Health literacy is defined as a multidimensional concept that encompasses the ability to access, evaluate and understand health-related information, thereby enabling the capacity to make informed decisions about health [13]. In the past, health education focused more on individuals’ ability to choose positive health behaviours as a means of disease prevention. However, this approach has been limited today, and a comprehensive approach that addresses not only individual but also social and environmental effects has been adopted to promote healthy lifestyle behaviours.

According to the model developed by Nutbeam in the 2000s, health literacy encompasses not only the ability to understand information effectively but also the capacity to critically evaluate the potential benefits of health information and take informed action in response to it. In this context, it is possible to express the contextual roots of health literacy as skills and individual capacity. Nutbeam examines the health literacy model at three levels [14]. The first level of this model, functional health literacy, reflects the outcome of traditional health education, which focuses on communicating basic information about health risks and how to utilize the health system. The second level, interactive health literacy, focuses on developing personal skills in a supportive environment and enhancing individuals’ capacity to act independently with the knowledge they possess. The third level, critical health literacy, reflects cognitive and skill development outcomes to support effective social and political action as well as individual action. This level of health literacy not only benefits the individual but also contributes significantly to the benefit of society [15].

Therefore, universities play a critical role in the multidimensional development of students’ health literacy capacity and in supporting healthy lifestyle behaviours [16]. The time spent at university is a period in which individuals experience significant changes in their health-related behaviours and attitudes. The knowledge and habits acquired during this period have the potential to affect not only the student’s own health but also the health of the family and community in the future [17].

However, according to the World Health Organization, inadequate health literacy has numerous negative consequences. The main effects of this situation include the adoption of unhealthy lifestyles and behaviours, inadequate use of disease prevention and early detection services, difficulties in self-management of chronic diseases, problems in adherence to medication, problems in understanding communication with health professionals, increased health care costs and deepening of existing health inequalities [18].

A study examining health literacy and related health behaviours found that health literacy was insufficient in the Jordanian population. In the same study, education level, age, living area and chronic diseases were identified as significant predictors of health literacy [19].

According to health literacy measurements conducted in sub-Saharan African countries, significant differences exist in health literacy rates among countries. In this context, 8.51% in Niger and 63.89% in Namibia have high health literacy levels. It is also stated that the majority of low- and middle-income countries are unable to measure health literacy or its impact on health objectively. This inadequacy constitutes an important shortcoming in comparing health literacy estimates across countries. It is also noted that the importance of health literacy as an independent concept has not yet been comprehensively explored in most low- and middle-income countries [20].

Somalia is ranked among low-income countries. The World Health Organization’s 2021–2025 plan and the Somalia Country Cooperation Strategy emphasize that health problems in Somalia are currently and will remain at an alarming level in the future. Poor hygiene and sanitation conditions are a critical factor in the recurrence and spread of diseases. The country is struggling with infectious diseases, maternal and neonatal mortality, and malnutrition, while also facing a growing burden of non-communicable diseases such as diabetes, obesity and cancer. Only 1.3% of the GDP, which amounted to USD 4.9 billion in 2019, is allocated to health, and per capita public health expenditure remains below USD 1 in 2020. Youth access to health services is very limited in the country (75% of the population is under the age of 30), and the weak tax system in the federal government structure severely restricts health investments [21].

A review of the existing literature reveals that there are no studies examining the relationship between health literacy and healthy lifestyle behaviours, particularly in low-income countries such as Somalia. This situation represents a significant gap in the development of health policies and education programs in these regions. This study aims to fill this critical gap in the existing literature by addressing the relationship between healthy lifestyle behaviours and health literacy levels among university students in Mogadishu. The findings of this research will provide important data to understand the health behaviours of youth in Somalia, and the data can contribute to shaping health policies more effectively and developing health education strategies.

## 2. Methods

### 2.1. The Study Design, Universe and Sample

This descriptive, cross-sectional study was conducted between 1 and 23 April 2024 in a university delivering education in Turkish in the field of health sciences in Mogadishu, Somalia. The study population comprised 425 students registered at the university, and the sample was composed of 219 health sciences students who agreed to participate in the research. The clinical trial number was not applicable in this study.

### 2.2. Ethical Approval

Before starting the study, written permission via email was obtained from the authors of the questionnaires that were planned to be used. Permission was obtained from the Research Committee of the institution where the study was conducted and approval was granted by the Non-Interventional Research Ethics Committee of Recep Tayyip Erdoğan Hospital, Mogadishu, Somalia (decision no: 959, dated: 30 March 2024).

### 2.3. Data Collection Tools

The data collection tools used were a demographic data form, the Healthy Lifestyle Behaviours Scale II (HLBS II) and the Turkish version of the European Health Literacy Scale (HLS-EU-TR). All the students participating in the research provided written informed consent. The data were collected through face-to-face interviews, and completing the questionnaire took an average of 15 min.

#### 2.3.1. Healthy Lifestyle Behaviours Scale-II

The Healthy Lifestyle Behaviours Scale was originally developed by Walker et al. (1987) as a 52-item, four-point Likert-type scale [22]. The Turkish validity and reliability study of the scale was conducted by Bahar et al. (2008) [23]. The scale consists of six subdimensions: health responsibility, physical activity, nutrition, spiritual growth, interpersonal relations and stress management. It is scored on a 4-point Likert scale, with response options ranging from “never” (1 point) to “regularly” (4 points). The total score ranges from a minimum of 52 to a maximum of 208. All items on the scale are positively worded, and higher scores indicate a more positive evaluation of healthy lifestyle behaviours [23]. The original scale demonstrated a Cronbach’s alpha coefficient of 0.92, while in the present study, it was found to be 0.93.

#### 2.3.2. European Health Literacy Scale (HLS-EU-TR)

The HLS-EU-TR is the Turkish adaptation of the European Health Literacy Survey Questionnaire (HLS-EU-Q). The scale was developed by the European Health Literacy Survey Consortium [24]. It is a self-report instrument designed to assess health literacy in individuals aged 15 and older. Validity and reliability studies of the Turkish version of the HLS-EU-TR were conducted by Abacıgil et al. in 2019 [25]. The conceptual framework includes three health-related domains of treatment, disease prevention and health promotion, as well as information processing stages related to health decision-making and actions: accessing, understanding, appraising and applying information [25]. For ease of calculation, the total score is standardized using a formula to range between 0 and 50. In this scoring system, 0 indicates the lowest and 50 the highest level of health literacy. The formula is shown as follows: Index = (mean score − 1) × (50/3). The Cronbach’s alpha coefficient of the scale is 0.95, and it was 0.97 in this study.

### 2.4. Data Analysis

Data obtained in the study were analysed statistically using SPSS vn. 23.0 software (Statistical Package for the Social Sciences). The homogeneity of the scores of both groups was analysed with the Kolmogorov−Smirnov test, Levene’s test and Box’s M test. Continuous variables were stated as mean ± standard deviation values, and categorical data were presented as numbers and percentages. The Pearson chi-squared test was used to compare categorical variables. The Student’s *t*-test and one-way ANOVA test were applied to independent groups to determine differences between the results of the measured variables. Pearson correlation coefficient (r) was used to examine the direction and strength of linear relationships between continuous variables. Pearson’s r was preferred due to the data meeting the assumptions of linearity and normal distribution. In the evaluation of correlation coefficients, coefficients between 0.10 and 0.29 were considered low, those between 0.30 and 0.49 were considered moderate, and those of >0.50 were considered a high degree of relationship [26]. Multiple Linear Regression (MLR) analysis was used to examine the relationship between the dependent variable and multiple independent variables. Prior to analysis, key MLR assumptions were checked, including linearity, absence of multicollinearity (VIF < 10), normal distribution of residuals (Q-Q plot and the Shapiro−Wilk test), homoscedasticity (the Breusch−Pagan test) and independence of errors (the Durbin−Watson test). Outliers were assessed using Cook’s distance. For all the analyses, a *p*-value of less than 0.05 was accepted to be statistically significant.

## 3. Results

Evaluation was conducted on 219 students, comprising 86.3% females and 13.7% males with a mean age of 20.91 ± 2.2 years. Of these, 92.7% were single, 58% were nursing students, and 29.7% were in the second year of study. The sociodemographic data of the study participants are shown in Table 1.

The total mean score of the Healthy Lifestyle Behaviours Scale II (HLBS II) was 127.54 ± 23.46 (min = 79, max = 208). The students’ average score from the Turkish version of the European Health Literacy Scale (HLS-EU-TR) was 30.33 ± 8.17, and they scored at problematic-borderline levels in treatment and service (42.5%), disease prevention (37.4%), health promotion (36.1%) and HLS-EU-TR Total Score (43.4%).

The results of the comparisons of the healthy lifestyle behaviours and health literacy scale of the students according to sociodemographic characteristics are shown in Table 2. The mean points of the physical activity subscale were determined to be higher for male students than for female students (t = −2.221, *p* = 0.014), and the spiritual development subscale mean points were statistically significantly higher for female students than for male students (t = 2.221, *p* = 0.027).

When the HLBS II mean points were examined according to year of study, a statistically significant difference was seen in the health responsibility, physical activity, nutrition and total HLBS II points (*p* = 0.000, *p* = 0.018, *p* = 0.010 and *p* = 0.007, respectively), with increases in these points as the year of study increased (*p* < 0.05).

The study participants who were single obtained statistically significantly higher points than married students in the HLS-EU-TR disease prevention and health development subscales and total scale points (*p* = 0.038, *p* = 0.048 and *p* = 0.034, respectively).

The evaluations of the relationships between the healthy lifestyle behaviours and health literacy scale of the students are presented in Table 3. A positive correlation at a low-moderate level was determined between healthy lifestyle behaviours and the HLS-EU-TR treatment and service points (r = 0.220, *p* = 0.001; r = 0.231, *p* = 0.001; r = 0.226, *p* = 0.001; r = 0.322, *p* ≤ 0.001; r = 0.300, *p* ≤ 0.001; r = 0.290, *p* ≤ 0.001; r = 0.336, *p* ≤ 0.001, respectively). With the exception of nutrition, a positive, moderate and low-level correlation was determined between healthy lifestyle behaviours and the HLS-EU-TR subscale of disease prevention (r = 0.167, *p* = 0.013; r = 0.149, *p* = 0.028; r = 0.125, *p* = 0.066; r = 0.250, *p* ≤ 0.001; r = 0.181, *p* = 0.007; r = 0.231, *p* = 0.001; r = 0.231, *p* = 0.001, respectively). There was found to be a positive, low, and very low level relationship between healthy lifestyle behaviours and the HLS-EU-TR subscale of health development (r = 0.237, *p* ≤ 0.001; r = 0.206, *p* = 0.002; r = 0.177, *p* = 0.009; r = 0.264, *p* ≤ 0.001; r = 0.193, *p* = 0.004; r = 0.250, *p* ≤ 0.001; r = 0.285, *p* ≤ 0.001, respectively). A positive correlation at a low-moderate level was determined between healthy lifestyle behaviours and the HLS-EU-TR total points (r = 0.230, *p* = 0.001; r = 0.215, *p* = 0.001; r = 0.193, *p* = 0.004; r = 0.308, *p* ≤ 0.001; r = 0.247, *p* ≤ 0.001; r = 0.284, *p* ≤ 0.001; r = 0.313, *p* ≤ 0.001, respectively).

Regression analysis was applied to the factors affecting the healthy lifestyle behaviours of the students (Table 4). The regression analysis conducted to determine the cause-and-effect relationship between health literacy and healthy lifestyle behaviours was found to be significant (F = 120.196; *p* < 0.001). A total of 13.1% of the variation in healthy lifestyle behaviours was explained by the HLS-EU-TR Treatment and Services subdimension and grade level (R^2^ = 0.131). The HLS-EU-TR Treatment and Services subdimension and grade level positively contributed to the level of healthy lifestyle behaviours (ß = 0.373; ß = 0.164).

## 4. Discussion

The results of this study demonstrated the relationship between the healthy lifestyle behaviours and levels of health literacy of health sciences students in Mogadishu, Somalia. The mean of the total scores obtained for the HLBS II was found to be 127.54 ± 23.46 and the mean of the total scores obtained for the HLS-EU-TR was 30.33 ± 8.17, and it was determined that the results obtained in all subdimensions (treatment and services, disease prevention and health development) and total scale scores of HLS-EU-TR were at problematic-borderline levels. In the comparisons made according to gender, the physical activity points of male students were higher, whereas female students obtained higher scores for spiritual development. As the year of study increased, an increase was observed in the health responsibility, nutrition and physical activity subdimensions and in the HLBS II total scores. The single students were determined to obtain higher points than the married students in the disease prevention and health development subdimensions and in the HLS-EU-TR total scale points. Positive correlations were found between the HLBS II and all dimensions of the HLS-EU-TR, and as a result of regression analysis, it was determined that 10% of the change in healthy lifestyle behaviours was explained by the HLS-EU-TR treatment and services subdimension and 5% of the change in healthy lifestyle behaviours was explained by the year of study. This study has addressed an important gap in the literature and is the first to present information specific to young people in Mogadishu, Somalia, which is classified as a low-income country [27].

The mean total points obtained in the HLBS-II by the Mogadishu university students were compared with data in the literature. A previous study conducted on first-year university students reported the mean points of the HLBS-II to be 129.01 ± 17.42 [28]. In another study conducted on university students to determine the effect of healthy lifestyle behaviours on smoking, the mean point of the HLBS-II was found to be 128.22 ± 24.89 [29]. In a study published in 2024 evaluating the HLBS-II mean scores of health sciences students, it was determined as 131.13 ± 20.33 [19]. The maximum possible score on the HLBS-II is 208. Higher mean scores indicate an increase in healthy lifestyle behaviours. The healthy lifestyle behaviours of university students in Mogadishu, Somalia, are consistent with the results reported in the literature. Given that the university where the study was conducted offers education in the field of health sciences, this result may be parallel to other studies.

The HLS levels of the vast majority of the university students in this study were determined to be at the borderline of being problematic. In a previous study that evaluated the health literacy levels of university students in health and social fields reported that 59% had insufficient or borderline problematic health literacy [30]. Another study found that more than 45% of adolescents and young adults had borderline health literacy levels [31]. Based on Nutbeam’s theoretical framework, health literacy levels that are at the problematic level cannot provide either individual or societal benefits [15]. Furthermore, as stated by the World Health Organization, low health literacy is associated with numerous negative outcomes [18]. There may be numerous factors (such as economic, governmental, and traditional and cultural differences) [21] that could contribute to the problematic health literacy scores of these students studying in Mogadishu, Somalia, and further studies are needed to identify these factors. Considering the benefits of both individuals and society, the fact that students are university students and receive health sciences education can be turned into an advantage by incorporating developmental courses into the curriculum, and health literacy can be improved.

The current study results of the HLBS II subscales showed that male students had higher physical activity scores and female students obtained statistically significantly higher points in the spiritual development subdimension. This finding was consistent with the results of a previous study of 334 university students, in which male students obtained higher physical activity points [32]. In contrast, some other studies have shown no gender-based difference in healthy lifestyle behaviours [33]. While the research results appear consistent with the literature, life is challenging for women and girls in Somalia. Somalia ranks fourth lowest in gender equality globally [34]. The interest shown by women in Somalia in spiritual health information suggests that cultural and social contexts may play a role. Gender disparities may allow men to access more information about physical health issues, while women may have more access to spiritual health information. These findings suggest that health policies and interventions should be tailored to gender. Future research should utilise both qualitative and quantitative methods to better understand gender health literacy gaps.

In the current study where evaluations were made according to the year of study, the health responsibility points were seen to be higher in the fourth- and second-year students compared to in the first-year students, the physical activity and nutrition scores were higher in the fourth-year students than in those in the first and third years, and the overall HLBS-II scores were determined to be higher in the fourth-year students than in those in the first year. It has been reported in the literature that as the year of study increased for nursing students, the HLBS-II points increased in parallel [5]. The increase in health responsibility scores in the second- and fourth-year students suggests that theoretical and practical lessons developed health knowledge and the students gave greater importance to their own health. The increase in physical activity and nutrition scores observed in the fourth-year students shows that the components of a healthy lifestyle were better understood over time. This situation demonstrates the impact of health education, and the high total HLBS-II scores of fourth-grade students also support the cumulative effect of education. This in turn supports the fact that positive behavioural changes are directly proportional to the period of education.

Compared to the students who were married, the single students in this study were determined to have obtained higher values in disease prevention and health development subdimensions and in the total HLS-EU-TR score. This was seen to differ from the results of previous studies in the literature that have reported that marital status did not affect levels of health literacy [35,36]. Families in Somalia are usually large, and therefore, family responsibilities, lack of time and economic burdens of married students may result in them having less time to spend in improving preventative health-related behaviours. The fact that the single students had greater knowledge, especially on the subject of disease prevention, and exhibited preventative health behaviours more often could have been due to their more productive use of time to access health information, participate in health-related activities and thereby benefit from preventative healthcare services. In this context, it is important that health education programs are planned taking into consideration the special circumstances of married students and that interventions are developed to increase the health literacy of this group.

The results of this study provide important insights into the relationship between healthy lifestyle behaviours and health literacy. There is a positive relationship between healthy lifestyle behaviours and the HLS-EU-TR treatment and services subscale. The treatment and services subscale of health literacy refers to the ability to access information on medical or clinical matters, understand medical information, interpret and evaluate medical information, make informed medical decisions and follow medical recommendations [37]. These skills are positively correlated with the healthy lifestyle behaviour subscales of health responsibility, physical activity, nutrition, spiritual development, interpersonal relationships and stress management. Developing healthy lifestyle behaviours can contribute to health literacy both individually and societally.

A positive correlation was noted between healthy lifestyle behaviours and the disease prevention subscale of the HLS-EU-TR. The disease prevention subscale refers to the ability to access information about health risk factors, understand and interpret information about risk factors, interpret and evaluate information about risk factors, and make informed decisions to protect against health risk factors [37]. These skills are not solely related to nutrition, which is one of the subscales of healthy lifestyle behaviours.

Similarly, there is a correlation between healthy lifestyle behaviours and the health promotion subscale of the HLS-EU-TR. The health promotion subscale refers to the ability to regularly update and understand health determinants in the social and physical environment, interpret and evaluate information about health determinants in the social and physical environment, make informed decisions about health determinants in the social and physical environment, and also engage in collective action [37].

Our research demonstrates a relationship between all subscales, healthy lifestyle behaviours and the HLS-EU-TR. This finding is significant given that health literacy among Somali university students is borderline problematic. Studies from low-income countries are quite limited in the literature, and because there are no data from Mogadishu, Somalia, the comparability of the findings is quite limited. However, the findings suggest that encouraging healthy lifestyle behaviours may be important for improving the health literacy of university students receiving health education in Mogadishu, Somalia. Furthermore, our findings indicate that health literacy increases with increasing education level. It can be argued that structuring education is crucial for increasing health literacy. This can help develop individuals and skills that form the framework of health literacy and, thus, benefit society.

This study has several limitations. First, the study is limited to data obtained from a single institution, which may affect the generalizability of the results. Due to the cross-sectional design, a cause-and-effect relationship between variables cannot be established; only the current situation can be determined. Considering these limitations, future studies using multicenter measurement methods are recommended.

## 5. Conclusions

Our research shows that the level of health literacy among university students studying health sciences in Mogadishu, Somalia is borderline problematic. As students’ years of education increase, healthy lifestyle behaviour scores increase, male students have higher physical activity subscale scores and female students have higher spiritual development subscale scores. Single students were found to have higher scores on the disease prevention and health promotion subscales and the total HLS-EU-TR score compared to married students. A positive correlation exists between healthy lifestyle behaviours and the HLS-EU-TR subscales. In Mogadishu, Somalia, designing or incorporating educational programs structured around the Nutbeam model and considering demographics such as gender and marital status to improve health literacy could contribute to health literacy and healthy lifestyle behaviours, both individually and societally.

## Figures and Tables

**Table 1:** Sociodemographic characteristics of students (n = 219).

Characteristics	Mean ± SD	
Age	20.91 ± 2.2	
Height	162.54 ± 10.76	
Weight	57.76 ± 10.91	
	**Number (n)**	**Percentage (%)**
**Gender**		
Female	189	86.3
Male	30	13.7
**Marital Status**		
Married	16	7.3
Single	203	92.7
**Department**		
Nursing	127	58.0
Midwifery	57	26.0
Emergency Aid and Disaster Management	35	16.0
**Class**		
1st Class	70	32.0
2nd Class	65	29.7
3rd Class	56	25.6
4th Class	28	12.8

**Table 2:** Comparison of participants’ sociodemographic characteristics, healthy lifestyle behaviours and health literacy scale.

Healthy Lifestyle Behaviours								Health Literacy Scale			
Variables	Health Responsibility	Physical Activity	Nutrition	Spiritual Development	Interpersonal Relationships	Stress Management	Total	Treatment and Services	Disease Prevention	Health Promotion	Total
**Gender**											
Female	21.1 ± 5.47	16.93 ± 5.31	20.47 ± 4.67	25.8 ± 5.36	22.86 ± 4.81	20.44 ± 4.13	127.5 ± 23.57	30.66 ± 8.55	30.01 ± 9.54	30.02 ± 9.15	30.23 ± 8.24
Male	21.46 ± 4.42	19.2 ± 4.35	21.46 ± 4.33	23.43 ± 5.83	21.76 ± 4.47	20.43 ± 3.66	127.76 ± 23.57	32.38 ± 8.46	30.05 ± 8.51	30.33 ± 10.02	30.92 ± 7.81
t/*p*	0.348/0.728	−2.221/**0.014**	−1.091/0.277	2.221/**0.027**	1.170/0.243	−0.14/0.989	−0.056/0.955	−1.024/0.307	−0.200/0.984	−170/0.865	−428/0.669
**Marital Status**											
Married	21.18 ± 5.36	18.25 ± 4.86	21.37 ± 5.08	25.54 ± 5.47	21.56 ± 5.13	19.5 ± 3.74	126.56 ± 22.47	27.46 ± 11.71	25.35 ± 11.69	25.67 ± 11.35	26.16 ± 10.4
Single	21.14 ± 5.35	17.16 ± 5.27	20.54 ± 4.6	24.68 ± 5.66	22.8 ± 4.74	20.51 ± 4.09	127.62 ± 23.58	31.17 ± 8.22	30.39 ± 9.11	30.41 ± 9.0	30.66 ± 7.91
t/*p*	−0.029/0.977	−0.798/0.426	−0.685/0.494	0.600/0.549	1.001/0.318	0.963/0.337	0.173/0.863	1.678/0.095	2.084/**0.038**	1.987/**0.048**	2.136/**0.034**
**Department**											
EADM	21.88 ± 5.98	18.54 ± 6.09	21.22 ± 4.3	26.17 ± 6.31	22.85 ± 4.61	20.77 ± 4.42	131.45 ± 26.15	31.91 ± 7.78	29.44 ± 8.43	30.21 ± 7.85	30.52 ± 7.13
Nursing	20.91 ± 5.24	17.32 ± 5.36	20.43 ± 4.92	25.11 ± 5.35	22.71 ± 4.84	20.64 ± 4.1	126.98 ± 23.83	30.55 ± 8.96	29.86 ± 9.72	29.62 ± 9.82	30.01 ± 8.61
Midwifery	21.22 ± 5.17	16.26 ± 4.2	20.61 ± 4.17	25.87 ± 5.23	22.61 ± 4.77	19.78 ± 3.75	126.38 ± 20.92	31.06 ± 8.09	30.75 ± 9.29	30.89 ± 8.78	30.92 ± 7.85
F/*p*	0.461/0.631	2.106/0.124	0.398/0.672	0.716/0.490	0.028/0.972	1.007/0.367	0.590/0.555	0.354/0.702	0.255/0.775	0.428/0.652	0.253/0.777
**Class**											
1st Class ^a^	19.35 ± 4.5	16.42 ± 4.73	19.77 ± 4.43	24.34 ± 5.18	21.77 ± 4.43	31.01 ± 8.87	121.64 ± 20.15	29.63 ± 8.02	28.55 ± 9.08	29.05 ± 9.43	29.07 ± 7.85
2nd Class ^b^	21.78 ± 5.17	17.36 ± 4.73	20.84 ± 4.54	26.26 ± 5.57	23.13 ± 4.88	31.01 ± 8.87	130.24 ± 23.14	31.8 ± 8.71	31.51 ± 9.3	31.40 ± 8.98	31.57 ± 8.29
3rd Class ^c^	21.16 ± 5.73	16.73 ± 5.75	20.12 ± 4.75	25.78 ± 5.39	22.66 ± 4.62	29.15 ± 9.62	126.17 ± 23.65	30.79 ± 9.09	29.55 ± 9.04	29.35 ± 9.85	29.89 ± 8.41
4th Class ^d^	24.14 ± 5.39	20.0 ± 5.81	23.1 ± 4.37	25.89 ± 5.97	24.17 ± 5.34	28.47 ± 11.63	138.75 ± 27.45	32.18 ± 8.31	31.15 ± 10.77	30.89 ± 8.14	31.41 ± 8.07
F/*p*	6.300/**0.000** **b,d > a**	3.445/**0.018** **d > a,c**	3.880/**0.010** **d > a,c**	1.569/0.198	1.991/0.116	1.233/0.299	4.127/**0.007** **d > a**	0.972/0.407	1.310/0.272	0.919/0.433	1.270/0.286

^a,b,c,d^ post hoc analysis.

**Table 3:** Relationship between participants’ healthy lifestyle behaviours and health literacy scale.

	Health Literacy Scale							
	Treatment and Services		Disease Prevention		Health Promotion		Total	
**Healthy Lifestyle Behaviours**	**r**	***p***	**r**	***p***	**r**	***p***	**r**	***p***
**Health Responsibility**	0.220	0.001	0.167	0.013	0.237	<0.001	0.230	0.001
**Physical Activity**	0.231	0.001	0.149	0.028	0.206	0.002	0.215	0.001
**Nutrition**	0.226	0.001	0.125	0.066	0.177	0.009	0.193	0.004
**Spiritual Development**	0.322	<0.001	0.250	<0.001	0.264	<0.001	0.308	<0.001
**Interpersonal Relationships**	0.300	<0.001	0.181	0.007	0.193	0.004	0.247	<0.001
**Stress Management**	0.290	<0.001	0.231	0.001	0.250	<0.001	0.284	<0.001
**Total**	0.336	<0.001	0.231	0.001	0.285	<0.001	0.313	<0.001

Pearson correlation test.

**Table 4:** Effect of health literacy on healthy lifestyle behaviours (n = 219).

Covariate	ß	SE	t	*p*	95% Confidence Interval	
					Lower	Upper
**Constant**		6.427	14.152	<0.001	78.289	103.627
**HLS-EU-TR** **Treatment and Services**	0.373	0.445	2.303	0.022	0.148	1.902
**HLS-EU-TR** **Health Promotion**	0.234	0.437	1.361	0.175	−0.267	1.457
**HLS-EU-TR** **Disease Prevention**	−0.093	0.257	−0.903	0.368	−0.738	0.274
**HLS-EU-TR** **Total**	−0.242	0.771	−0.903	0.368	−2.215	0.823
**Class**	0.164	1.452	2.577	**0.011**	0.880	6.605

F = 9.168, R^2^ = 0.131, *p* < 0.001; multiple linear regression analysis; **HLS-EU-TR**—European Health Literacy Turkish.

## Data Availability

The data presented in this study are available on request from the corresponding author. Our data can be shared upon request and cannot be shared directly for the following reasons: To protect the confidentiality of study participants, our ethics committee approval does not permit public sharing of the data; The data are subject to data protection regulations in Somalia; However, anonymized data can be shared through the corresponding author upon reasonable request.
